# Mental Fatigue Degree Recognition Based on Relative Band Power and Fuzzy Entropy of EEG

**DOI:** 10.3390/ijerph20021447

**Published:** 2023-01-13

**Authors:** Xin Xu, Jie Tang, Tingting Xu, Maokun Lin

**Affiliations:** School of Communications and Information Engineering, Nanjing University of Posts and Telecommunications, Nanjing 210003, China

**Keywords:** mental fatigue, EEG, N-back task, CEEMD, ICA, feature extraction, ensemble learning, XGBoost

## Abstract

Mental fatigue is a common phenomenon in our daily lives. Long-term fatigue can lead to a decline in a person’s operational functions and seriously affect work efficiency. In this paper, a method that recognizes the degree of mental fatigue based on relative band power and fuzzy entropy of Electroencephalogram (EEG) is proposed. The N-back experiment was used to induce mental fatigue in subjects, and the corresponding EEG signals were recorded during the experiment. A preprocessing method based on complementary ensemble empirical modal decomposition (CEEMD) and independent component analysis (ICA) was designed to remove noise from the raw EEG signal. The relative band power feature, which has been used extensively in fatigue recognition studies, was extracted from the EEG signals. Meanwhile, fuzzy entropy, a feature commonly used in attention recognition, was also extracted for fatigue recognition, based on previous findings that an increase in fatigue is accompanied by a decrease in attention. The two features were fed into an extreme gradient boosting (XGBoost) classifier to distinguish three different degrees of fatigue, which resulted in an average accuracy of 92.39% based on data from eight subjects. The promising results indicate the effectiveness of the proposed method in mental fatigue degree identification.

## 1. Introduction

The definition of mental fatigue, first proposed by Grandjean in 1979, considers fatigue to be a physical state, a transitional state between wakefulness and sleep [1]. It is manifested by a reluctance to exert energy, reduced efficiency and alertness and impaired mental performance [2]. With the development of modern technology, the pace of life is becoming faster and faster, and the pressure from work and study is increasing. Prolonged cognitive tasks that require continuous concentration inevitably lead to fatigue, resulting in undesired symptoms such as delayed reactions, dizziness, and nausea that seriously affect people’s lives. Therefore, it is necessary to identify and analyze different levels of fatigue in order to help reduce its negative effects.

Electromyography (EMG) and electrocardiography (ECG) are two commonly used measures for fatigue detection. Wu et al. studied the effect of musical rhythm on the subjective and objective fatigue of runners at different exercise intensities using EMG [3]. Butkevičiūtė used ECG for the identification of fatigue [4]. Electroencephalography (EEG), which can directly measure the neurophysiological activity in the human brain and its corresponding changes caused by fatigue [5], is also considered as a reliable method for fatigue detection. 

Previous research has reported some promising results in the field of fatigue recognition based on EEG signals. Zhang et al. calculated the power of the EEG signals in four frequency bands as features to distinguish between fatigue and wakefulness states. An accuracy of 81.07% was achieved in a multi-task learning framework [6]. Trejo et al. demonstrated an increase in power spectral density (PSD) of α waves and θ waves at the onset of fatigue. Based on the PSD features, a kernel partial least squares classifier was able to achieve 98.80% accuracy in fatigue state identification [7]. Wang et al. used common spatial pattern (CSP) to extract features from 14 EEG channels and reported a 90% accuracy with support vector machine (SVM) [8].

Preprocessing is an essential part of EEG signal processing that plays an important role in removing noise and improving the signal-to-noise ratio (SNR) of the signal. It is generally believed that the noise in EEG signals exists at frequencies above 30 Hz, so using a low-pass filter to remove signals higher than 30 Hz is the traditional method of noise removal, but the processing of signals by this method is relatively rough. In order to further improve the signal quality and lay a good foundation for following processing, a signal denoising method based on complementary ensemble empirical modal decomposition (CEEMD) and independent component analysis (ICA) is designed in this paper, with CEEMD responsible for decomposing the signal into several frequency bands and ICA responsible for separating out the noise components in the expectation of achieving more detailed denoising. CEEMD decomposes the signal into several intrinsic mode functions (IMFs) and a residual signal without modal aliasing and auxiliary noise residuals [9,10]. ICA is a common signal processing method that can decompose signals into a few independent components (ICs) [11].

The feature extraction of EEG signals is also a key factor affecting the effectiveness of the model. The fatigue degree can eventually be accurately recognized only when the selected features contain valid information that helps to classify. EEG features associated with band power (e.g., power spectral density, band power ratio) have been widely used in the study of fatigue degree recognition. Hendrawan et al. extracted power percentage features for each EEG segment and used the LDA algorithm to recognize fatigue with an accuracy of 92.82% [12]. Zeng et al. used band power ratios (β/θ and α/θ) as features and domain-adversarial neural networks (DANN) as classifiers in their fatigue recognition study, achieving a recognition accuracy of 91.63% [13]. These studies have suggested that features related to the band power of EEG signals can be useful for fatigue recognition. Therefore, in this paper, the relative band power of EEG signals is employed as a feature for fatigue recognition. Relative band power is defined as the band power ratio of δ, θ, α and β frequency bands relative to the total frequency band.

A number of studies have shown a correlation between fatigue and attention. Van der Linden et al. suggested that mental fatigue can also be manifested as a decrease in attention [14]. Azarnoosh et al. found that mental fatigue leads to a decrease in overall brain activity and a decrease in attention as one of the regular activities of the brain in response to incoming stimuli [15]. Boksem et al. showed that mental fatigue reduces attention levels [16]. Meanwhile, fuzzy entropy is a commonly used EEG feature that has shown its effectiveness in attention recognition [17,18]. Therefore, this study considers the introduction of features that have been proven effective in attention recognition studies, and fuzzy entropy satisfies this condition. With the help of the relationship between attention level and fatigue degree, effective features of attention recognition (fuzzy entropy) are added to the fatigue recognition task as a way to aid the recognition of fatigue degree.

In this work, eight subjects were invited to participate in an N-back experiment to induce fatigue, while the corresponding EEG signals were recorded through the process. A denoising method based on CEEMD and ICA was designed to remove noise from the raw EEG signals. Relative band power and fuzzy entropy were extracted as features of the EEG signal. Based on these features, the extreme gradient boosting (XGBoost) algorithm was employed to classify three different degrees of mental fatigue.

The paper is organized as follows. Section 2 describes the methods of EEG preprocessing, feature extraction, and classification. Section 3 describes the experiment results. Section 4 is the discussion, and Section 5 concludes this paper.

## 2. Materials and Methods

### 2.1. N-Back Experiment

The N-back experiment, which is one of the most common experimental paradigms in brain load elicitation experiments, was first proposed by Kirchner in 1958 to study the short-term memory capacity of people of different ages for rapidly changing information [19].

**Figure 1:**
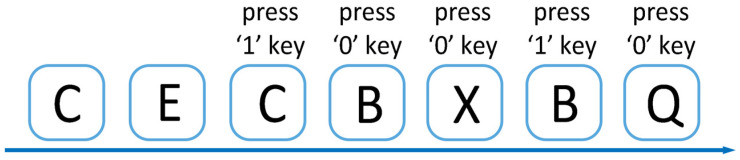
2-back Experiment.

At the beginning of the experiment, a cross symbol appears in the center of the screen to remind subjects to focus. During the experiment, a capital letter will appear randomly on the screen, and the subject needs to determine whether the current letter is consistent with the letter presented N items ago, and if it is consistent, press the “1” key, otherwise press the “0” key. The 1-back experiment requires the subject to determine whether the current letter is the same as the previous letter, while the 2-back experiment requires a comparison with the letter two items ago. The process of the 2-back experiment is shown in Figure 1.

### 2.2. EEG Data Acquisition

Studies have demonstrated that 30 min of continuous 1-back or 2-back tasks can effectively induce fatigue [20]. In order to obtain fatigue-related EEG signals, eight healthy subjects (five males and three females) were invited to participate in the N-back experiment. The subjects were all university students aged 20 to 26 years old, with normal vision. The students had no history of neurological disorders and had not taken any medication within 24 h prior to the experiment.

The experiment was divided into three stages: Stage 1, where the EEG signals were recorded in the subject’s calm state for 5 min; Stage 2: where the subject completed a 30 min 1-back task and recorded the EEG signals in the last 5 min of the task; Stage 3, where the subject completed a 30 min 2-back task and recorded the EEG signals in the last 5 min. The EEG signals acquired in the experiment contained 19 channels, the electrodes were arranged according to the international 10–20 system, the right and left earlobes were selected as reference electrodes, and the sampling rate was 512 Hz.

After each stage of the experiment, subjects were guided to fill out a subjective fatigue scale, as shown in Table 1. Subjects were also instructed to rate their degree of attention on a scale of 1 to 10, with higher scores representing more confused thinking and distraction.

### 2.3. Preprocessing

Preprocessing has two main tasks: segmentation of the acquired raw EEG signal for further processing, and removal of noise from the raw EEG.

**Figure 2:**
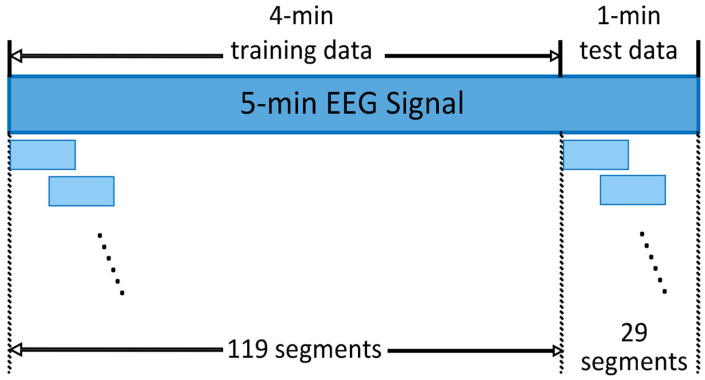
EEG Data Segment.

The EEG signals were first segmented as shown in Figure 2. For each of the three experimental stages, a 5 min EEG signal was recorded, the first 4 min of which were used for training, and the last 1 min for testing. Both training and test data were divided into segments of 4 s with a 2 s overlap between two adjacent segments. To avoid data leakage, there was no overlap between the last segment of the training data and the first segment of the test data. For each 5 min EEG signal, a total of 119 segments of training data and 29 segments of test data were obtained.

Each segment of the EEG signal then needed to be denoised. In this paper, a method based on CEEMD and ICA was designed for EEG denoising. It is first desired to decompose the EEG signal into several frequency bands and then analyze which of them contains more noise. The noise-containing band should be denoised and then the signal of each frequency band is reconstructed to obtain the denoised EEG signal. 

**Figure 3:**
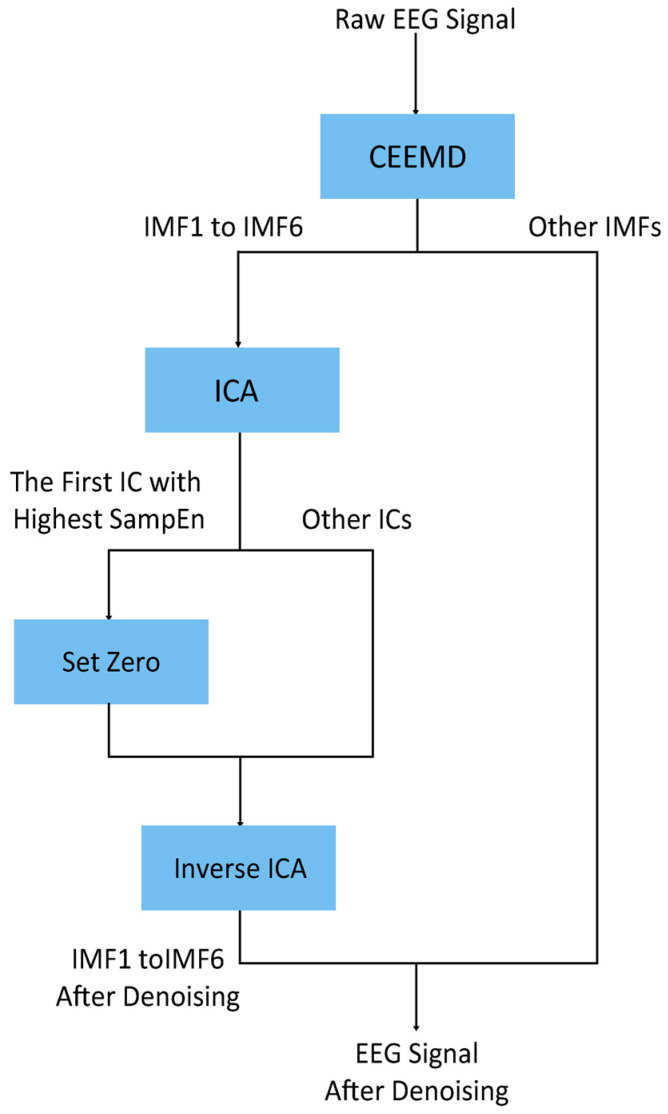
Signal Preprocessing Process.

According to the frequency range, EEG is divided into five frequency bands: delta, theta, alpha, beta, and gamma, but this way of division is rather rough and is not suitable for a more detailed analysis of where the noise lies in the frequency range. Wavelet analysis can decompose the signals into multiple frequency bands in a more detailed way, but its effect is affected by the wavelet function used, so it needs to analyze which wavelet function to use to obtain the best results. CEEMD, on the other hand, performs an adaptive decomposition of the signal into a number of IMFs, each representing a frequency component, without any other analysis. Therefore, this paper used CEEMD to decompose the EEG signal. The value of sample entropy is positively correlated with the noise power [21]. That is, a larger sample entropy indicates a more complex signal, indicating that the signal contains more noise; conversely, a smaller sample entropy indicates a higher degree of autocorrelation, suggesting that the signal contains less noise. Therefore, this paper used sample entropy to find the IMFs that contained more noise. In conventional preprocessing methods, IMFs that are considered to contain noise are usually discarded directly, which usually results in the loss of some useful signal components. To improve this problem, a further step with ICA was performed on the noisy IMFs. More specifically, the IMFs containing noise were processed by ICA to obtain a number of ICs, and then the sample entropy of each IC was calculated to find the ICs that contained more noise. The first IC with the highest value of sample entropy was regarded as a noise-containing component and set to zero. Then, the inverse ICA was performed on all ICs to obtain the noise-removed IMFs. Finally, the denoised IMFs and the IMFs considered to contain no noise were reconstructed, and the reconstructed signal was the preprocessed denoised signal. The preprocessing process is shown in Figure 3.

**Figure 4:**
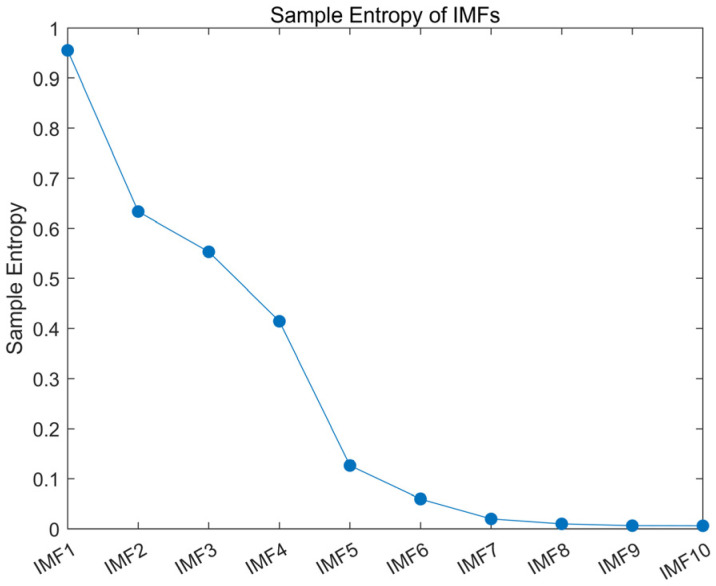
Sample Entropy of IMFs.

The raw EEG signal was decomposed into several IMFs using CEEMD, and the sample entropy of each IMF was calculated. Figure 4 shows the calculated sample entropy of each IMF after the signal was decomposed by CEEMD. The sample entropy of IMFs decreased in order, and the first six IMFs had larger sample entropy values, while the remaining IMFs had smaller sample entropy. After several repeated experiments, the acquired EEG signals all showed this characteristic. Therefore, the first six IMFs of the raw signal were considered to contain more noise, while the rest of the IMFs did not. Then, the ICA was performed on the first six IMFs to obtain a number of ICs, and the IC with the highest sample entropy value was set to zero; then, the inverse ICA was applied to obtain the denoised IMF1–IMF6. The denoised EEG signal was obtained by reconstructing all IMFs.

### 2.4. Feature Extraction

In this work, the relative band power and the fuzzy entropy of EEG signals were extracted as features for fatigue recognition. Relative band power has been widely used in fatigue recognition studies. We assumed here that this feature would play an important role in recognizing fatigue degrees. Considering that an increase in fatigue level is often accompanied by a decrease in attention level, this paper introduced fuzzy entropy, which is a feature commonly used for attention level recognition, to help improve the accuracy of fatigue level recognition. The details of these features are described as follows. 

Relative band power (RBP) is the ratio of the power of the EEG signal in a particular frequency band to the total power. The PSD of the EEG signal was first estimated using the Welch method, and then the power percentage of the different frequency bands was calculated according to (1).

(1) $${\mathrm{RBP}}_{\alpha }=\frac{\sum_{f_{\alpha }}P(f)}{\sum_{{f=f}_{1}}^{f_{2}}P(f)}$$

where P(f) represents the PSD of the EEG signal, f_1_ and f_2_ represent the minimum and maximum frequencies of the EEG signal, respectively, and $f_{\alpha }$ represents the frequency range of α band. Similarly, we could calculate the power percentage of the β, θ and δ frequency bands [22]. In particular, the δ band had a frequency range between 0 and 3 Hz, and the θ band was between 4 and 7 Hz, while the α and β bands were located between 8 and 13 Hz and 14 and 30 Hz, respectively.

Fuzzy entropy is used to measure the complexity and irregularity of a time series. It is calculated as follows [23]. Let the signal sequence containing N samples be {u(i): 1 ≤ i ≤ N}; this sequence forms a set of m dimensional vectors $x_{i}^{m}$ as shown in (2).

(2) $$x_{i}^{m}=\left\{u\left(i\right),u\left(i+1\right),\ldots ,u\left(i+m-1\right)\right\}\;{-\;u}_{0}\left(i\right),\;i=1,2,\ldots ,L\;-\;m$$

where u(i),u(i+1),…, u(i+m−1) is the i-th point to the i + m − 1 point of sequence u(i), and $u_{0}\left(i\right)$ is their mean value.

(3) $$u_{0}\left(i\right)=\frac{1}{m}\overset{m-1}{\underset{j=0}{\sum }}u\;(i+j)$$

$d_{\mathrm{ij}}^{m}$ is defined as the distance between vector $x_{i}^{m}$ and $x_{j}^{m}$, and its value represents the maximum difference between the corresponding elements of the two vectors.

(4) $$d_{\mathrm{ij}}^{m}=d\left[x_{i}^{m}{,x}_{j}^{m}\right]=\underset{k\in \left(0,m-1\right)}{\max}\left\{|u\left(i+k\right)\;{-\;u}_{0}\left(i\right)\;-\;(u\left(j+k\right)\;{-\;u}_{0}\left(j\right))|\right\}$$

where i, j = 1~N − m and i ≠ j.

The fuzzy entropy function u($d_{\mathrm{ij}}^{m}$, n, r) is used to define the similarity of vectors $x_{i}^{m}$ and $x_{j}^{m}$, as shown in (5).

(5) $$D_{\mathrm{ij}}^{m}=u\left(d_{\mathrm{ij}}^{m},n,r\right){=e}^{\frac{d_{\mathrm{ij}}^{m}}{r}}$$

where n and r denote the gradient and width of the fuzzy function boundary. The function $\emptyset^{n}$ is defined according to (6).

(6) $$\emptyset^{n}\left(n,r\right)=\frac{1}{n-m}\overset{N-m}{\underset{i=1}{\sum }}\left(\frac{1}{N-m-1}\overset{N-m}{\underset{j=1,\;j\neq i}{\sum }}D_{\mathrm{ij}}^{m+1}\right)$$

Ultimately, fuzzy entropy is defined as (7).

(7) $$\mathrm{FuzzyEn}\left(m,n,r\right)=\;\underset{N\to \infty }{\lim}\left[\ln\emptyset^{n}\left(n,r\right)-\ln\emptyset^{n+1}\left(n,r\right)\right]$$

The raw EEG signal was divided into 4 s segments after pre-processing, and each segment contained 19 channels. The above five features were calculated for each channel, so a total of 95 feature values were obtained for each segment.

### 2.5. Classification

XGBoost is currently one of the most advanced algorithms in the field of ensemble learning. It follows the general modelling process of the Boosting algorithm, i.e., building the base estimators in turn, computing a loss function based on the output of the previous base estimators, and adaptively influencing the construction of the next base estimators. The final output of the ensemble algorithm is influenced by all of the base estimators. Specifically, suppose the XGBoost algorithm has a total of K base estimators (typically decision tree); for a certain sample x_i_, the final output H(x_i_) is expressed as (8).

(8) $${H(x}_{i})=\overset{K}{\underset{k=1}{\sum }}{\eta f}_{k}{(x}_{i})$$

where f_k_(x_i_) denotes the output of sample x_i_ on the k-th base estimator, and η is the learning rate, which is a hyperparameter in the XGBoost algorithm. In addition, as the iteration progresses, the ensemble output result for each sample is continuously computed. The output of sample x_i_ when building the k-th base estimator is shown in (9).

(9) $$H_{k}{(x}_{i}{)=H}_{k-1}{(x}_{i}{)+\eta f}_{k}{(x}_{i})\;$$

Unlike other Boosting algorithms, XGBoost adaptively influences the creation of weak evaluators by fitting pseudo-residuals. Specifically, before each new base estimator is built, the first-order derivative $g_{\mathrm{ik}}$ and the second-order derivative $h_{\mathrm{ik}}$ of the current loss function with respect to the output are computed, as shown in (10) and (11).

(10) $$g_{\mathrm{ik}}=\;{\left[\frac{\partial l\left(y_{i},H\left(x_{i}\right)\right)}{\partial H\left(x_{i}\right)}\right]}_{H\left(x_{i}\right){=H}_{k-1}\left(x_{i}\right)}$$

(11) $$h_{\mathrm{ik}}=\;{\left[\frac{\partial^{2}l\left(y_{i},H\left(x_{i}\right)\right)}{\partial H^{2}\left(x_{i}\right)}\right]}_{H\left(x_{i}\right){=H}_{k-1}\left(x_{i}\right)}$$

where $l\left(y_{i},H\left(x_{i}\right)\right)$ is the loss function, and $y_{i}$ is the true label of sample $x_{i}$. Then, the current pseudo-residuals are

(12) $$r_{\mathrm{ik}}=-\frac{g_{\mathrm{ik}}}{h_{\mathrm{ik}}}\;$$

When building a new base estimator, the model needs to fit the pseudo-residuals $r_{\mathrm{ik}}$ rather than the true label value $y_{i}$ of the sample. This approach allows XGBoost to ensure that the loss function is minimized at each iteration.

When performing the classification task, XGBoost feeds the ensemble output into the Softmax function and obtains the classification probability. The final classification decision is made by choosing the class with the highest probability. In this work, we employed XGBoost to identify fatigue degrees due to its fast modeling speed and high resistance to over-fitting. 

In this paper, the relative band power of the δ, θ, α and β bands and the fuzzy entropy were extracted as features of the EEG signals. The above features were calculated for each of the three fatigue degrees, and the feature matrix was fed into the XGBoost classifier. Considering the inter-subject variability, a subject-specific model was used in this paper, i.e., a separate classification model was trained for each subject. EEG signals representing each of the three fatigue degrees were recorded in each of the three stages of the data acquisition experiment, with each recording lasting 5 min. According to the proposed data segmentation method, each 4 s segment was defined as a sample. Each subject had 119 training samples and 29 test samples at each fatigue degree, and each subject recorded EEG at three different fatigue degrees. This led to a total of 357 training samples and 87 test samples for each subject.

## 3. Experiment and Results

### 3.1. Experiment

In this study, the relevant EEG signals were acquired and processed according to the method described in Section 2. 

**Figure 5:**
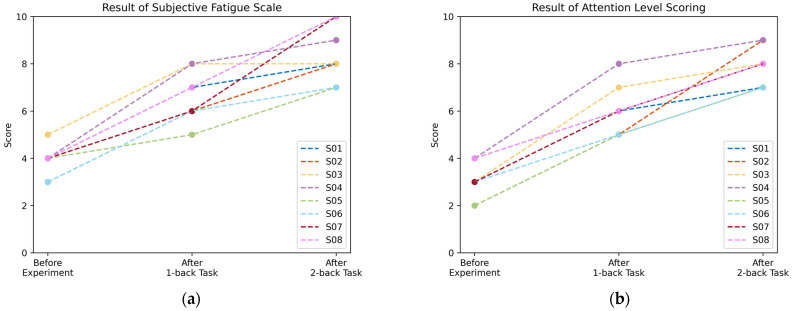
Subjective Scale Scores, (**a**) Fatigue, (**b**) Attention Level.

First, subjects were invited to perform the N-back task to induce mental fatigue while EEG signals were collected (as described in Section 2.2). Finally, each subject acquired three EEG signals of 5 min in duration, representing three different degrees of fatigue. In the data acquisition process, subjects were guided to fill out a subjective fatigue scale and rate their degree of attention on a scale of 1 to 10. The results of the subjective scale for the eight subjects are shown in Figure 5. As the experiment progressed, the subjective fatigue scale scores increased, which is consistent with the trend of deepening mental fatigue. In addition, subjects’ concentration and clarity of thought decreased, suggesting that the increase in fatigue was accompanied by a decrease in attention. Based on these results, the EEG signals collected during the three stages of the experiment are valid data for fatigue recognition.

**Figure 6:**
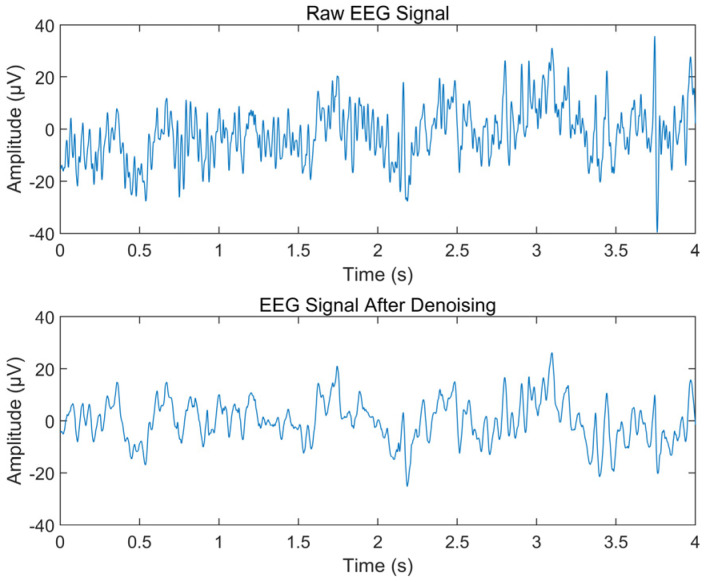
Time Domain Waveforms Before and After Denoising.

**Figure 7:**
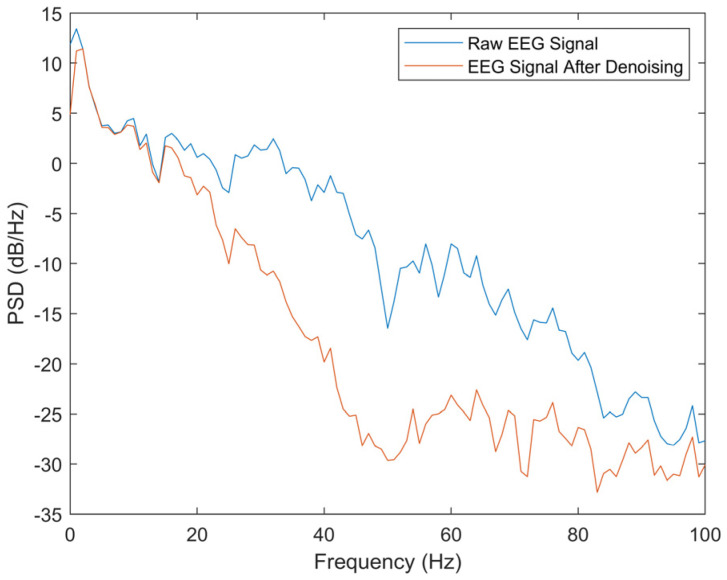
PSD of EEG Before and After Denoising.

Then, the signal is segmented and denoised using the method described in Section 2.3. For each 5-min EEG signal, a total of 119 segments of training data and 29 seg-ments of test data were obtained. Figure 6 shows the time domain waveforms before and after denoising of a 4-s EEG signal. This method effectively removed the high frequency noise from the raw EEG signal without losing significant signal components. The power spectral density of EEG before and after denoising (Figure 7) also shows that the artifact components with frequency greater than 30 Hz are effectively suppressed.

Then, the relative band power of the four frequency bands of EEG and the fuzzy entropy are calculated. As described in Section 2.4 and Section 2.5, there were 357 samples in the training set and 87 samples in the test set for each subject, and each sample had a total of 95 values of the features. 

Finally, the 357 × 95 feature matrix of each subject was used to train the subject-specific XGBoost classifier and the model was tested using the 87 × 95 feature matrix.

### 3.2. Evaluation Metrics

Accuracy is a common evaluation metric for classification problems and is defined as the percentage of correctly classified samples in the test set. Once the prediction results are obtained, one of the three degrees of fatigue is considered as a positive case and the others as negative cases. Then, three confusion matrices are drawn based on the classification results. Precision and recall are calculated separately on each confusion matrix, denoted as $\left(P_{1}{,R}_{1}\right)$, $\left(P_{2}{,R}_{2}\right)$ and $\left(P_{3}{,R}_{3}\right)$. Then, the average precision and recall are computed to obtain the macro-precision (macro-P), the macro-recall (macro-R) and the macro-F1-score (macro-F1).

(13) $$\mathrm{macro-P}=\frac{1}{3}\overset{3}{\underset{i=1}{\sum }}P_{i}$$

(14) $$\mathrm{macro-R}=\frac{1}{3}\overset{3}{\underset{i=1}{\sum }}R_{i}$$

(15) $$\mathrm{macro-F}1=\frac{2\;\times \;\mathrm{macro-P}\;\times \;\mathrm{macro-R}}{\;\mathrm{macro-P}+\mathrm{macro-R}}$$

### 3.3. Results

For each subject, the XGBoost classifier was trained on the training data following a five-fold cross-validation procedure, and the hyperparameters of the model were adjusted with its average classification accuracy as the optimization target. After training, the model was tested and the classification accuracy, macro-precision, macro-recall, and macro-f1-score of the classifier on the test data were calculated. Finally, the model classified the fatigue degree of eight subjects with an average accuracy of 92.39%, macro-precision of 92.67%, macro-recall of 92.39% and macro-f1-score of 92.31%. The specific evaluation metrics are shown in Table 2. The classification performance of XGBoost classifier was also compared with that of SVM, random forest and AdaBoost classifiers, as shown in Table 3. Experimental results showed that the XGBoost classifier outperformed the other classifiers.

## 4. Discussion

### 4.1. Comparison with Other Preprocessing Methods

In this paper, an EEG preprocessing method based on CEEMD and ICA was proposed. To further illustrate the effectiveness of the method, three other methods were used to preprocess the data in this paper separately. Both features, relative band power and sample entropy, were also calculated, and the XGBoost classifier was also used to compare their classification performance. The three preprocessing methods were low-pass filtering, wavelet threshold denoising, and CEEMD, where low-pass filtering used a low-pass filter with a passband of less than 30 Hz, wavelet threshold denoising used a 4-layer decomposition of the db4 wavelet with a soft threshold function and a fixed threshold, and CEEMD decomposed the signal into several IMFs and reconstructed it after discarding the first six IMFs. Table 4 shows the performance of the classification for the three fatigue degrees using different preprocessing methods. The results show that the classification results obtained with the preprocessing method of this paper were better.

### 4.2. Comparison with Other Methods

Table 5 shows a comparison of the method proposed in this paper with some of the existing methods. Similarly to our study, the studies in Table 5 also designed their own experiments to induce mental fatigue in subjects, extracting multiple features of EEG signals and recognizing the degree of fatigue using certain classification methods. It should be noted that the results listed in the table were from different EEG data, so the comparison does not directly indicate that the method proposed in this study is superior to other existing methods. However, it can still show to a certain extent that our method can reach a good level in the related field.

### 4.3. Limitations of the Current Study

This paper proposed a fatigue recognition method based on EEG signals that includes four parts: data acquisition, preprocessing, feature extraction and classification. Experimental results demonstrated that our method is able to recognize three different degrees of mental fatigue with high accuracy. However, there were certain limitations of the current study that need to be improved in further studies.

First, the method may suffer from feature redundancy. Relative band power of four frequency bands and fuzzy entropy were selected as EEG features. Therefore, five feature values needed to be calculated for each segment of EEG. Since there were 19 channels per segment of EEG, there were a total of 95 feature values per sample. This led to a computationally intensive algorithm, which is not conducive to practical applications, especially for real-time detection of fatigue degree. The use of feature selection algorithms to select the features that contribute most to the classification helps to reduce the redundancy of features, and the design of channel optimization algorithms to find the most important channels can also further reduce the computational effort. 

Second, how the features of the EEG signal affect the classification results remains to be further studied. In this paper, a variety of features were extracted, and the classification results showed that these features together were effective in distinguishing different fatigue levels. However, it is unclear whether there is a significant difference between the features at three fatigue levels. For example, the same method obtained an accuracy of 100.00% on subject S01, but only 83.91% on S03. Is this because one or more features did not differ significantly between the three levels of fatigue for S03? More in-depth analysis is needed to understand the effect of each feature on the classification results.

Admittedly, the method proposed in this paper is still not representative of the state-of-the-art. Designing a more rational experimental paradigm to more effectively induce mental fatigue in subjects, optimizing the preprocessing process to further improve signal quality, screening and using features that are valid across all subjects, and using a classification model with greater learning ability are all areas for improvement in this study. The resolution of these issues will contribute further to the practical application of fatigue recognition technology.

## 5. Conclusions

Analyzing mental fatigue EEG signals can identify different levels of fatigue, thereby helping to reduce the negative effects of fatigue. In this paper, we proposed a method to recognize three degrees of fatigue based on EEG signal analysis. Subjects were invited to participate in the N-back experiment, and the subjective scale demonstrated that the experiment was successful in inducing different degrees of fatigue. Raw EEG signals were preprocessed by CEEMD and ICA to effectively remove noise and retain as much useful signal as possible. The relative band power commonly used in fatigue recognition studies and the fuzzy entropy commonly used in attention recognition studies were selected as the features of EEG signals, and XGBoost classifier was used to classify the different degrees of mental fatigue. Experimental results show that the proposed method can recognize three different degrees of fatigue with high accuracy.

## Figures and Tables

**Table 1:** Subjective Fatigue Scale.

Fatigue Level	Symptom Description	Scoring
Vigilance	High attention span	1~2
Sobriety	Normal condition, no noticeable sensation	3~4
Mild Fatigue	Mild eye weakness	5~6
Moderate Fatigue	Slow reactions and dizziness	7~8
Heavy Fatigue	Headache and nausea	9~10

**Table 2:** Classification Evaluation Metrics.

Subject	Accuracy	Macro-P	Macro-R	Macro-F1
S01	100.00%	100.00%	100.00%	100.00%
S02	91.95%	92.73%	91.95%	91.89%
S03	83.91%	83.89%	83.91%	83.69%
S04	91.95%	92.10%	91.95%	91.97%
S05	90.80%	91.56%	90.80%	90.60%
S06	97.70%	97.74%	97.70%	97.70%
S07	86.21%	86.50%	86.21%	86.11%
S08	96.55%	96.88%	96.55%	96.54%
Average	92.39%	92.67%	92.39%	92.31%

**Table 3:** Comparison of Classification Performance of Different Classifiers.

Classifier	Accuracy	Macro-P	Macro-R	Macro-F1
SVM	87.07%	88.92%	87.07%	86.90%
Random Forest	89.80%	90.32%	89.80%	89.72%
AdaBoost	79.17%	79.08%	79.17%	77.73%
XGBoost	93.10%	92.39%	92.67%	92.31%

**Table 4:** Comparison of Classification Performance of Different Preprocessing Methods.

Preprocessing Method	Accuracy	Macro-P	Macro-R	Macro-F1
Low-pass Filter	89.66%	91.22%	89.66%	89.44%
Wavelet Threshold Denoising	88.94%	91.09%	88.94%	88.57%
CEEMD	64.80%	65.69%	64.80%	64.54%
This Work	93.10%	92.39%	92.67%	92.31%

**Table 5:** Comparison of Existing Methods.

Authors	Features	Classifier	Accuracy (%)
Nguyen et al. [24]	blink rate eye closure rate heart rate α and β band power	FLDA	79.2
Wu et al. [25]	PSD of δ, θ, α and β power ratio of (δ + θ)/(α + β), (α + θ)/β, θ/β, α/β	DCSAEN	81.5
Zeng et al. [13]	power ratio of β/θ and α/θ	DANN	91.63
Luo et al. [26]	band energy and relative energy of δ, θ, α and β	SVM	92.24
Liu et al. [27]	PSD of of δ, θ, α and β sample entropy	SRDA	90.875
This Work	relative band power fuzzy entropy	XGBoost	92.39

FLDA: Fisher’s linear discriminant analysis; DCSAEN: deep stacked contractive sparse autoencoder network; DANN: domain-adversarial neural network; SVM: support vector machine; SRDA: spectral regression discriminant analysis.

## Data Availability

The data presented in this study are available on request from the corresponding author. The data are not publicly available due to privacy restrictions.
